# Trends in growth and nutritional status of high school graduates in Hangzhou, China, 2011–2020

**DOI:** 10.1186/s12889-022-13253-2

**Published:** 2022-04-25

**Authors:** Xu Duan, Yi-nan Zhou, Yun Chen

**Affiliations:** 1 Department of Cardiology, Hangzhou First People’s Hospital, Hangzhou, China; 2 Hangzhou Education Examination Authority, Hangzhou, China

**Keywords:** Height, Body mass index, Thinness, Overweight, Obesity, Adolescents

## Abstract

**Background:**

During past decades, there was a positive trend in growth and nutrition status of adolescents in China, but there was significant regional disparity. The purpose of this study is to investigate the trends in growth and nutritional status of high school graduates in Hangzhou between 2011 and 2020.

**Methods:**

High school graduates (Grade 12) who finished the physical examination of the national college entrance examination between 2011 and 2020 (n=481,353)were included in this study. Data were obtained from the database of physical examination of the national college entrance exam. Height and weight were measured; body mass index (BMI) was calculated from height and weight. Thinness, overweight and obesity were defined according to the International Obesity Task Force criteria. For the vast majority of the high school graduates were 18 years old or nearly 18 years old, the cutoffs of 18 years were adopted. Those are 18.5, 25 and 30 kg/m^2^, for thinness, overweight and obesity respectively.

**Results:**

There was a significant growth trend in height, weight and BMI in both sexes (*P* < 0.001). Height increased by 1.80 cm in boys and 1.45 cm in girls. Weight increased by 4.62 kg in boys and 2.51 kg in girls. BMI increased by 1.09 kg/m^2^ in boys and 0.60 kg/m^2^ in girls. An increase trend was found in the prevalence of overweight and obesity in both sexes (*P* < 0.001). Overweight increased by 7.43% (from 9.05 to 16.48%) among boys and 4.05% (from 4.57 to 8.62%) among girls. Obesity increased by 3.85% (from 2.29 to 6.14%) among boys and 1.76% (from 0.64 to 2.40%) among girls. The prevalence of thinness fluctuated in both boys and girls, 12.42–15.59% among boys and 18.97–23.68% among girls. Boys had higher odds of overweight and obesity and lower odds of thinness than girls (*P* < 0.001).

**Conclusions:**

There is a positive trend in growth and nutritional status of high school graduates in Hangzhou. However, there is still a considerable prevalence of thinness, it indicates a double burden of undernutrition and overnutrition.

## Introduction

Growth and nutritional status in adolescence play an important role in the whole human life. Both undernutrition and overnutrition in adolescence are associated with adverse health consequences even in adulthood [1–6]. Undernutrition in adolescence not only negatively impacts growth and puberty development [1], but also elevates the risk of diseases, such as infection, fracture and so on [2, 3]. Overnutrition in adolescence increases risk of obesity in adulthood [4] and is associated with elevated long-term risk of cardiovascular diseases and all-cause mortality [5, 6].

The trends of growth and nutritional status of adolescents are associated with socioeconomic development [7–12]. In developed countries, the positive trend in growth has decelerated or flatten [7, 8], and the prevalence of overweight overtaking underweight becomes a more important public health problem [9, 10]. In China, given the dramatic socioeconomic development during past decades, there is a positive trend in growth and a shift from problem of undernutrition to overnutrition in adolescents [11–13]. Because of the regional variations in socioeconomic development across China, there is a significant regional disparity of nutritional status. The prevalence of stunting and thinness is higher in western provinces, such as Guizhou and Guangxi. Meanwhile, the prevalence of overweight is higher in the eastern coastal provinces and municipalities such as Tianjin and Beijing. Some provinces, such as Guangdong, Guangxi, Chongqing, and Sichuan, are facing a double burden of undernutrition and overnutrition [13].

Hangzhou is a developed city located in the southeast of China. During the last decade, the social economy of Hangzhou developed rapidly [14, 15]. We hypotheses that there was a positive trend of growth and nutritional status of adolescents in Hangzhou during the last decade. The present study investigated the trends in growth and nutritional status of high school graduates in Hangzhou between 2011 and 2020.

## Methods

### Study subjects

In China, every high school graduate who apply to college is required to take a physical examination before the national college entrance examination(Gaokao). The current study is based on the data of physical examination of high school graduates in Hangzhou.

A total of 481,353 students finished the physical examination of the national college entrance examination between 2011 and 2020. Data were exported from the database of physical examination of the national college entrance examination. 2 students were excluded because of obvious mistakes of height and weight, one with a height of 1 cm and the other with a height of 0 cm and a weight of 0 kg. And thus, a total of 481,351 students were finally included, 229,555 boys and 251,796 girls.

### Measurement and assessment

Height and weight were measured by trained doctors or nurses. Height was measured without shoes to the nearest 1 cm and weight was measured with light clothes to the nearest of 1 kg. Every student had a chance of remeasurement after the physical examination, if he or she questioned the results.

Body mass index(BMI) was calculated as weight in kilograms divided by the square of height in meters. Thinness, overweight and obesity were defined according to the International Obesity Task Force(IOTF) criteria [16, 17]. For the official age of entry into primary school is 6 years old in China, the vast majority of the high school graduates(Grade 12) were 18 years old or nearly 18 years old. Therefore, the cutoffs of 18 years were adopted. Those are 18.5, 25 and 30 kg/m^2^, for thinness, overweight and obesity respectively.

### Statistical analysis

Continuous variables including height, weight and BMI were expressed as mean and SD, differences between sexes were examined by the t-test. Linear regression was used to examine the trends in height, weight and BMI during the study period. Categorical variables including the prevalence of thinness, overweight and obesity were expressed as proportions and 95% confidence interval (CI), differences between sexes were examined by Pearson’s chi-squared test, odds ratio(OR) was calculated. Linear-by-linear association test was used to examine the trends in prevalence of thinness, overweight and obesity during the study period.

All statistical analyses were performed with the statistical software SPSS version 24. A two-sided *P* < 0.05 was considered statistically significant.

## Results

### Trends of anthropometric measurements

**Fig. 1:**
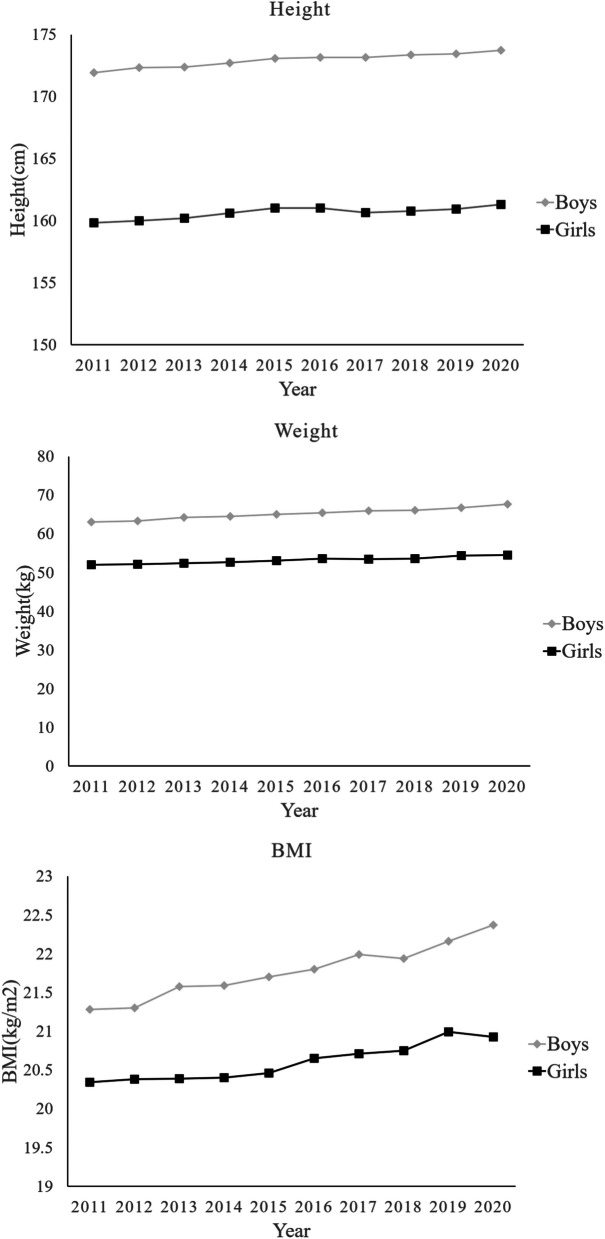
Trends in height, weight and BMI of high school graduates in Hangzhou

Table 1 and Fig. 1 presents the anthropometric measurements and trends by sex. There was a significant growth trend in height and weight in both sexes between 2011 and 2020(*P* < 0.001). Height increased by 1.80 cm (from 171.95 ± 5.85 to 173.74 ± 6.04 cm) in boys and increased by 1.45 cm(from 159.84 ± 5.38 to 161.30 ± 5.59) in girls, except a slight decrease in 2017. Weight increased by 4.62 kg (from 63.01 ± 10.53 to 67.63 ± 13.65 kg) in boys and increased by 2.51 kg (from52.00 ± 7.78 to 54.51 ± 9.86 kg) in girls, except a slight decrease in 2017.

**Table 1:** Anthropometric measurements of high school graduates in Hangzhou between 2011 and 2020

	n		Height(cm)				Weight(kg)				BMI(kg/m^2^)				
	Boys	Girls	Boys		Girls		Boys		Girls		Boys		Girls		*P*
			Mean	SD	Mean	SD	Mean	SD	Mean	SD	Mean	SD	Mean	SD	< 0.001
Total	229,555	251,796	172.93	5.91	160.62	5.49	65.18	11.87	53.18	8.33	21.77	3.62	20.59	2.94	< 0.001
2011	24,205	26,154	171.95	5.85	159.84	5.38	63.01	10.53	52.00	7.78	21.28	3.24	20.34	2.76	< 0.001
2012	24,488	26,587	172.35	5.77	159.99	5.35	63.36	10.70	52.21	7.57	21.30	3.24	20.38	2.66	< 0.001
2013	23,828	26,249	172.37	5.80	160.21	5.37	64.21	11.12	52.38	7.68	21.58	3.39	20.39	2.70	< 0.001
2014	23,115	25,972	172.71	5.84	160.62	5.42	64.48	11.34	52.66	7.83	21.59	3.44	20.40	2.77	< 0.001
2015	21,826	24,621	173.10	5.85	161.00	5.51	65.09	11.71	53.07	8.11	21.70	3.58	20.46	2.82	< 0.001
2016	21,305	24,277	173.18	5.89	161.00	5.52	65.47	11.75	53.57	8.30	21.80	3.58	20.65	2.93	< 0.001
2017	20,464	22,910	173.15	5.87	160.64	5.54	66.00	12.09	53.48	8.16	21.99	3.70	20.71	2.88	< 0.001
2018	21,929	24,433	173.38	5.89	160.77	5.49	66.05	12.20	53.66	8.49	21.94	3.70	20.75	3.01	< 0.001
2019	23,507	24,861	173.44	6.04	160.95	5.58	66.74	12.48	54.41	8.87	22.16	3.82	20.99	3.14	< 0.001
2020	24,888	25,732	173.74	6.04	161.30	5.59	67.63	13.65	54.51	9.86	22.37	4.20	20.93	3.53	< 0.001
P for linear regression			< 0.001		< 0.001		< 0.001		< 0.001		< 0.001		< 0.001

A significant trend of increase in BMI was found in both sexes between 2011 and 2020(*P* < 0.001). BMI increased by 1.09 kg/m2(from 21.28 ± 3.24 to 22.37 ± 4.20 kg/m2) in boys, except a slight decrease in 2018. It increased by 0.60 kg/m2(from 20.34 ± 2.76 to 20.93 ± 3.53 kg/m2) in girls, except a slight decrease in 2020. Boys had significantly higher BMI than girls(21.77 ± 3.62 kg/m^2^ vs 20.59 ± 2.94 kg/m^2^; *P* < 0.001).

### Trends of nutritional status

**Fig. 2:**
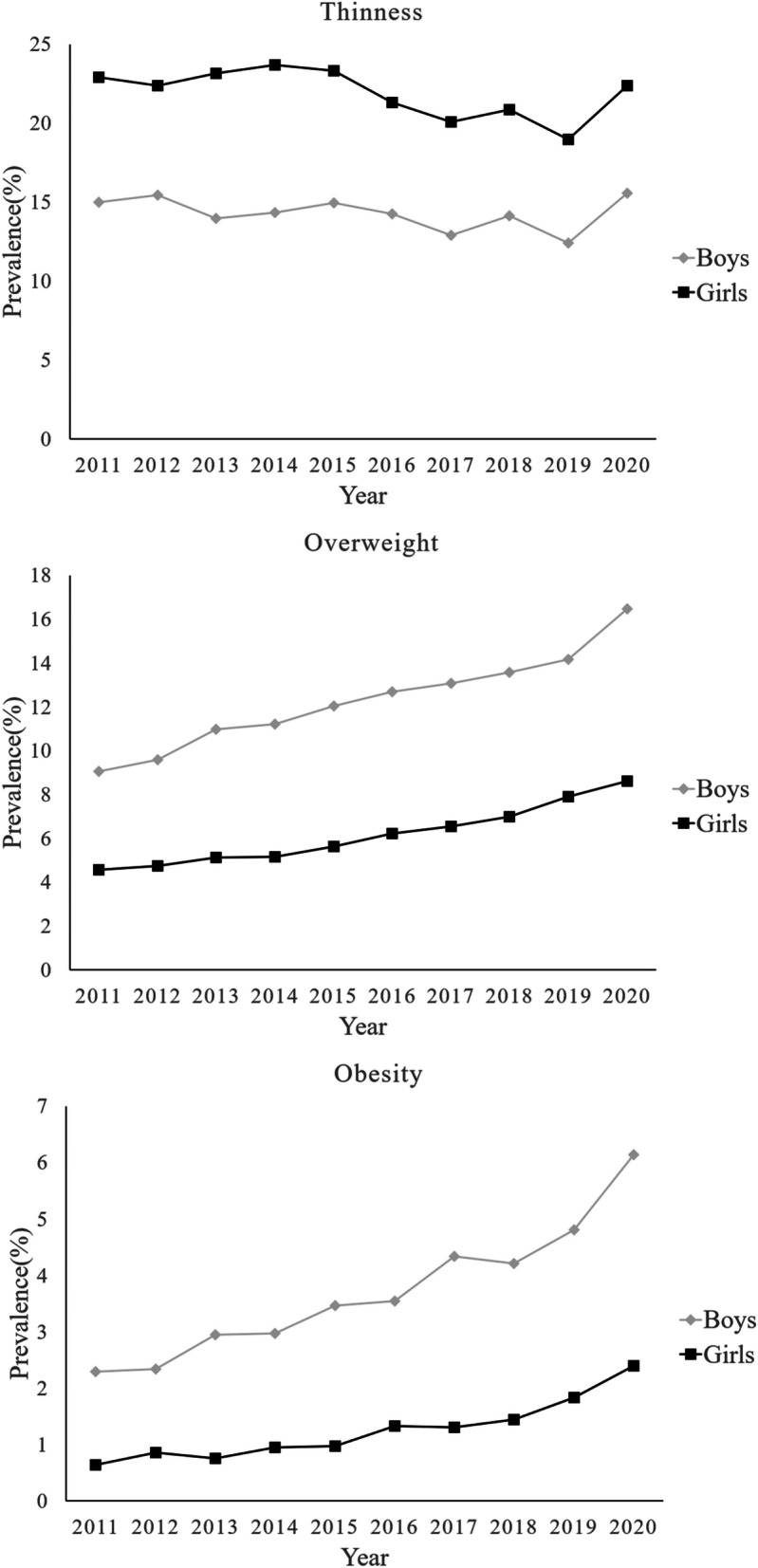
Trends in prevalence of thinness, overweight and obesity in high school graduates in Hangzhou

Table 2 and Fig. 2 presents the prevalence and trends of thinness, overweight and obesity by sex. The prevalence of overweight and obesity was 12.27 and 3.70% in boys and 6.13 and 1.24% in girls. Boys had higher odds of overweight(OR 2.14, 95%CI 2.10, 2.19) and obesity(OR 3.06, 95%CI 2.93,3.19) than girls. A positive trend was found in the prevalence of overweight in both sexes between 2011 and 2020 (*P* < 0.001). The prevalence of overweight increased by 7.43%(from 9.05 to 16.48%) in boys and increased by 4.05%(from 4.57 to 8.62%) in girls. There was a positive trend in the prevalence of obesity in both sexes between 2011 and 2020 (*P* < 0.001). The prevalence of obesity in boys increased by 3.85%(from 2.29 to 6.14%), except a slight decrease in 2018. The prevalence of obesity in girls increased by 1.76%(from 0.64 to 2.40%), except slight decreases in 2013 and 2017.

**Table 2:** Prevalence of thinness, overweight and obesity in high school graduates in Hangzhou between 2011 and 2020

	n		Prevalence of thinness (%)			Prevalence of overweight (%)			Prevalence of obesity (%)		
	Boys	Girls	Boys	Girls	*P*	Boys	Girls	*P*	Boys	Girls	*P*
Total	229,555	251,796	14.33 (14.19–14.48)	21.95 (21.79–22.11)	< 0.001	12.27 (12.13–12.40)	6.13 (6.03–6.22)	< 0.001	3.70 (3.62–3.78)	1.24 (1.20–1.28)	< 0.001
2011	24,205	26,154	14.99 (14.54–15.44)	22.93 (22.42–23.44)	< 0.001	9.05 (8.69–9.41)	4.57 (4.31–4.82)	< 0.001	2.29 (2.10–2.48)	0.64 (0.55–0.74)	< 0.001
2012	24,488	26,587	15.44 (14.99–15.90)	22.37 (21.87–22.87)	< 0.001	9.59 (9.22–9.96)	4.73 (4.48–4.99)	< 0.001	2.34 (2.15–2.53)	0.86 (0.75–0.97)	< 0.001
2013	23,828	26,249	13.99 (13.55–14.43)	23.17 (22.66–23.68)	< 0.001	10.97 (10.58–11.37)	5.13 (4.86–5.39)	< 0.001	2.95 (2.74–3.17)	0.75 (0.65–0.85)	< 0.001
2014	23,115	25,972	14.34 (13.89–14.79)	23.68 (23.16–24.20)	< 0.001	11.20 (10.79–11.60)	5.17 (4.91–5.44)	< 0.001	2.97 (2.75–3.19)	0.95 (0.84–1.07)	< 0.001
2015	21,826	24,621	14.97 (14.50–15.45)	23.32 (22.79–23.85)	< 0.001	12.03 (11.60–12.46)	5.63 (5.34–5.92)	< 0.001	3.46 (3.22–3.71)	0.97 (0.85–1.09)	< 0.001
2016	21,305	24,277	14.25 (13.79–14.72)	21.33 (20.81–21.84)	< 0.001	12.69 (12.24–13.14)	6.22 (5.92–6.52)	< 0.001	3.54 (3.29–3.79)	1.33 (1.19–1.48)	< 0.001
2017	20,464	22,910	12.91 (12.45–13.36)	20.09 (19.57–20.61)	< 0.001	13.07 (12.61–13.53)	6.54 (6.22–6.86)	< 0.001	4.34 (4.06–4.62)	1.30 (1.15–1.44)	< 0.001
2018	21,929	24,433	14.13 (14.59–13.67)	20.85 (20.34–21.36)	< 0.001	13.57 (13.12–14.02)	7.00 (6.68–7.32)	< 0.001	4.21 (3.94–4.47)	1.44 (1.30–1.59)	< 0.001
2019	23,507	24,861	12.42 (12.00–12.84)	18.97 (18.48–19.45)	< 0.001	14.17 (13.72–14.62)	7.89 (7.55–8.22)	< 0.001	4.81 (4.53–5.08)	1.83 (1.66–2.00)	< 0.001
2020	24,888	25,732	15.59 (15.14–16.04)	22.37 (21.86–22.88)	< 0.001	16.48 (16.02–16.94)	8.62 (8.27–8.96)	< 0.001	6.14 (5.85–6.44)	2.40 (2.21–2.58)	< 0.001
P for linear-by-linear association test			< 0.001	< 0.001		< 0.001	< 0.001		< 0.001	< 0.001

The prevalence of thinness was 14.33% in boys and 21.95% in girls in the whole sample. Boys had lower odds of thinness(OR 0.60, 95%CI 0.59,0.60) than girls. Although the prevalence of thinness significantly different among years(*p* < 0.001), there was no trend of increase or decrease between 2011 and 2020. It fluctuated between 12.42 and 15.59% in boys and between 18.97 and 23.68% in girls.

## Discussion

During the last century, a large progression in body height and weight was observed all over the world in both sexes, regardless of the country, climate, race or socioeconomic situation [7, 8, 18–20]. In developed countries, the increase of growth had decelerated or stopped since late twentieth century [18, 19, 21, 22]. In China, positive trend of growth was still observed at the early twenty-first century [12, 23], which was significant associated with the development of socioeconomic status and medical and health conditions. The positive trend may continue as the economic development and urbanization process of China [12, 23], or it may be plateaued like other developed countries [18, 22]. Given China’s vast territory, there are regional disparities of growth among different regions, which are attributed to not only difference of socioeconomic development, but also differences of genetic factor, geography, climate and so on [24]. In Hangzhou, the height of high school graduates in both sexes between 2011 and 2020 were lower than counterparts in Changzhou(another developed city in the southeast of China) between 2004 and 2011 [25].There were also lower than those of 17 years old adolescents in Beijing City(capital of China) in 2010 [26]. Even so, there was a positive trend in height of high school graduates in Hangzhou between 2011 and 2020. Previous studies have proved that the trend of height is significantly associated with socioeconomic development [12, 26]. Therefore, the positive trend of height in this study may attribute to the social-economic development of Hangzhou. The GDP per capital of Hangzhou in 2019(data of 2020 has not be available yet) was 152,465 RMB, nearly twice as much as 80,478 RMB in 2011; the urbanization rate in 2019 was 78.5%, higher than 73.9% in 2011 [14, 15]. In this study, the increment rate of height was higher in boys than in girls, which was similar to the results of other studies in China [23, 26]. But the exact mechanism deserves further study.

Overweight and obesity of children and adolescents is a public health challenge in both developing and developed countries [10, 27, 28]. The prevalence of overweight and obesity is associated with socioeconomic development, which is higher in developed counties than developing countries [27]. In some high-income countries, the rising trend in BMI of children and adolescents had plateaued at a high level [10]. In China, the prevalence of overweight and obesity in children and adolescents increased continuously during past decades [29, 30], but there were significant regional disparities. In Beijing, the prevalence of obesity in children and adolescents decreased slightly in both sexes between 2005 and 2010 [26]. In Changzhou, the prevalence of obesity in high school graduates increased slightly between 2004 and 2011, but the prevalence of overweight was stable in both sexes [25]. In our study, positive trend in the prevalence of overweight and obesity were observed in high school graduates between 2011 and 2020. It may be attributed to the continuous economic growth and urbanization of Hangzhou [14, 15]. As the development of economy and urbanization, the changes of life style result in increase of overweight and obesity in children and adolescents, including more secure food supply, more energy-dense diet, reduced energy expenditure in transportation, more inactive leisure time, and so on [11, 31, 32]. In addition, the academic burden of school-aged children and adolescents should be taken into account. In China, school-aged children and adolescents bear an increasing academic burden, especially high school students who are preparing for the college entrance exam(Gaokao) [33]. The academic burden is associated with insufficient physical activity and excessive screen time, those are proved to be risk factors of overweight and obesity [34–36].

Although overweight and obesity increase globally, underweight is still an important public health problem, especially in developing countries [10]. In China, although there was a nutritional transition from underweight to overweight and obesity during past 30 years, there was still a considerable proportion of thinness among children and adolescents. In 2014, the prevalence of thinness was 13.1% in Han(majority population in China) and 17.1% in ethnic minority children and adolescents aged 7–18 years, according to the IOTF criteria [37]. In our study, the prevalence of thinness among high school graduates was 14.33% in boys and 21.95% in girls, it is higher than the prevalence of overweight in both sexes. It indicated a double burden of undernutrition and overnutrition in high school graduates in Hangzhou. During the study period, the prevalence of thinness was fluctuated without significant decrease. Similar trend was observed in another study of high school graduates in Changzhou between 2004 and 2011 [25]. It indicated that thinness may be a long-term public health problem, even with the development of economy.

In China, boys have higher prevalence of overweight and obesity and lower prevalence of thinness than girls [37], except some ethnic minorities such as Mongol, Tibet, Uyghur and so no [38]. In our study, boys are more likely to be overweight and obese and less like to be thin, and the increment rate of overweight and obesity was higher in boys than girls. The sex disparity may be attributed to differences of cognition and behaviors between boys and girls. In China, girls have worse body esteem and are more likely to misperceive themselves as overweight [39, 40]. Therefore, girls are more prone to control weight. The 2015 Chinese national youth risk behavior surveillance suggested that girls were more likely to constrict dieting for losing weight and less like to have soft drinks frequently and to play computer games [41].

Because this study is a retrospective study based on the physical examination of the national college entrance examination. There are some limitations to this study. Firstly, data of exact age was not available. Therefore, thinness, overweight and obesity were defined according to the cutoffs of 18 years old, but not the exact age of every subject. Secondly, the measurement was carried out at several medical stations and the equipments were not unified. And the accuracy of height and weight was up to 1 cm and 1 kg. But the large sample size may compensate for the measurement error to some extent. Thirdly, this study didn’t exclude students who applied to college and took physical examination several times in different years. It may affect the independence of sample from years, although they were only a very small part of study population. Fourthly, urban and rural areas were not distinguished in this study, although the growth and nutrition status of adolescents are different between urban and rural areas [11, 24].

## Conclusions

This study demonstrates that there is a positive trend of growth and nutritional status of high school graduates in Hangzhou. However, there is still a considerable prevalence of thinness without significant decrease, especially in girls. It indicates a double burden of undernutrition and overnutrition in high school graduates in Hangzhou.

The results of Hangzhou add more understanding of growth and nutritional status of adolescents in China. Double duty actions and sex specific actions were needed for public health policies.

## Data Availability

Because the datasets analyzed during the current study are obtained from the database of physical examination of the national college entrance exam in Hangzhou, they are not publicly available but are available from the corresponding author on reasonable request.

## Abbreviations

BMI: Body mass index
CI: Confidence interval
OR: Odds ratio
